# Radiotherapy of abdomen with precise renal assessment with SPECT/CT imaging (RAPRASI): design and methodology of a prospective trial to improve the understanding of kidney radiation dose response

**DOI:** 10.1186/1471-2407-13-381

**Published:** 2013-08-10

**Authors:** Juanita Lopez-Gaitan, Martin A Ebert, Peter Robins, Jan Boucek, Trevor Leong, David Willis, Sean Bydder, Peter Podias, Gemma Waters, Brenton O’Mara, Julie Chu, Jessica Faggian, Luke Williams, Michael S Hofman, Nigel A Spry

**Affiliations:** 1 School of Physics, The University of Western Australia, Perth, Australia; 2 Department of Radiation Oncology, Sir Charles Gairdner Hospital, Perth, Australia; 3 Department of Nuclear Medicine, Sir Charles Gairdner Hospital, Perth, Australia; 4 Department of Radiation Oncology, Peter MacCallum Cancer Centre, Melbourne, Australia; 5 North West Cancer Centre, Tamworth, Australia; 6 School of Surgery, The University of Western Australia, Perth, Australia; 7 Centre for Cancer Imaging, Peter MacCallum Cancer Centre, Melbourne, Australia; 8 Department of Medicine and Pharmacology, The University of Melbourne, Melbourne, Australia; 9 School of Medicine and Pharmacology, The University of Western Australia, Perth, Australia

**Keywords:** Radiotherapy, Kidney, Functional imaging

## Abstract

**Background:**

The kidneys are a principal dose-limiting organ in radiotherapy for upper abdominal cancers. The current understanding of kidney radiation dose response is rudimentary. More precise dose-volume response models that allow direct correlation of delivered radiation dose with spatio-temporal changes in kidney function may improve radiotherapy treatment planning for upper-abdominal tumours.

Our current understanding of kidney dose response and tolerance is limited and this is hindering efforts to introduce advanced radiotherapy techniques for upper-abdominal cancers, such as intensity-modulated radiotherapy (IMRT). The aim of this study is to utilise radiotherapy and combined anatomical/functional imaging data to allow direct correlation of radiation dose with spatio-temporal changes in kidney function. The data can then be used to develop a more precise dose-volume response model which has the potential to optimise and individualise upper abdominal radiotherapy plans.

**Methods/design:**

The Radiotherapy of Abdomen with Precise Renal Assessment with SPECT/CT Imaging (RAPRASI) is an observational clinical research study with participating sites at Sir Charles Gairdner Hospital (SCGH) in Perth, Australia and the Peter MacCallum Cancer Centre (PMCC) in Melbourne, Australia. Eligible patients are those with upper gastrointestinal cancer, without metastatic disease, undergoing conformal radiotherapy that will involve incidental radiation to one or both kidneys. For each patient, total kidney function is being assessed before commencement of radiotherapy treatment and then at 4, 12, 26, 52 and 78 weeks after the first radiotherapy fraction, using two procedures: a Glomerular Filtration Rate (GFR) measurement using the ^51^Cr-ethylenediamine tetra-acetic acid (EDTA) clearance; and a regional kidney perfusion measurement assessing renal uptake of ^99m^Tc-dimercaptosuccinic acid (DMSA), imaged with a Single Photon Emission Computed Tomography / Computed Tomography (SPECT/CT) system. The CT component of the SPECT/CT provides the anatomical reference of the kidney’s position. The data is intended to reveal changes in regional kidney function over the study period after the radiotherapy. These SPECT/CT scans, co-registered with the radiotherapy treatment plan, will provide spatial correlation between the radiation dose and regional renal function as assessed by SPECT/CT. From this correlation, renal response patterns will likely be identified with the purpose of developing a predictive model.

**Trial registration:**

Australian New Zealand Clinical Trials Registry: ACTRN12609000322235

## Background

Radiotherapy has a significant role to play in the management of cancers of the upper abdomen, but high dose radiotherapy has been considerably limited by potential adverse normal tissue responses, in particular that of the kidneys. Because of their proximity to organs such as the pancreas, stomach and oesophagus, incidental radiation dose to the kidneys is frequently unavoidable when planning radiotherapy treatment. Our current understanding of kidney dose response and tolerance to radiation is rudimentary. This limits the delivery of a higher dose to the tumour and also the introduction and optimisation of new techniques to treat these upper gastrointestinal cancers, such as intensity modulated radiation therapy (IMRT).

Until recently, estimation of radiation-induced kidney damage was based on analysis of very basic radiotherapy data by Emami et al [1]. The Quantitative Analyses of Normal Tissue Effects in the Clinic (QUANTEC) review collated results of relevant studies that have been undertaken so far [2]. There have been previous key studies that have addressed the effects of partial kidney irradiation. In 1973, Thompson et al [3] reported the occurrence of clinical kidney toxicity several years after radiotherapy. Willet et al [4] found a dependence on the percentage of the volume being irradiated with a decrease in creatinine clearance. More recently, Kost et al [5] in 2002, used sequential scintigraphy to assess impairment of renal function and correlated it with the radiation dose distribution. Jansen et al [6] observed a progressive decrease in renal function in gastric cancer patients after postoperative radiotherapy. Studies in pigs [7,8] have shown that the response is dependent on whether one or both kidneys are irradiated and that the functional response depends on the compensatory response of the non-irradiated kidney.

A review of the preceding studies and the QUANTEC report [2] has highlighted several key points which have influenced the design of the RAPRASI study protocol:

– Kidneys have a late response to radiation (between 3 and 18 months).

– The functional response is dependent on the percentage of the kidney volume being irradiated and the dual nature of this organ influences their response.

– There is limited quantitative data to support dose-volume models for the kidney. Studies are required with collection of data that would allow correlating regional kidney function over time with the radiation dose distribution received in the radiotherapy treatment.

– The glomerular filtration rate (GFR) should be used to assess the toxicity of the kidney, as recommended by the National Kidney Foundation [9]. Early changes in renal perfusion and GFR correlate with an increased risk of late toxicity [2] and chronic injury is unlikely if changes in GFR and perfusion are not detected by 24 months after irradiation [10,11].

– Significant technological advances in imaging offer the possibility to study late responding normal tissues using quantitative functional imaging instead of a conventional histological analysis [12].

GFR can be estimated using serum creatinine levels and estimating equations but in order to achieve a higher degree of accuracy, a ^51^Cr-ethylenediamine tetra-acetic acid (EDTA) clearance measurement should be used. On the other hand, scintigraphy with Single Photon Emission Computed Tomography (SPECT) can provide greater detail regarding the relative distribution of kidney function; kidney perfusion as indicated by uptake of ^99m^Tc-dimercaptosuccinic acid (DMSA), imaged with scintigraphy, has been shown to correlate with relative regional function [13,14]. Sequential GFR measurements using the ^51^Cr-EDTA clearance and SPECT imaging undertaken before, during and after radiotherapy, allows observation of regional functional changes in the kidney over time and these changes can be correlated with the radiation dose delivered during radiotherapy.

In order to quantify the relationship between radiation dose delivery and kidney response, methods are required that will allow the registration of the estimated three-dimensional (3D) radiotherapy dose distribution to the regional changes in kidney function, indicated by changes in DMSA uptake [15]. In a SPECT/CT system [16], both Computed Tomography (CT) and SPECT imaging is undertaken on the one device with the patient in a single position. This minimises any influence of patient motion or physiological changes between the two imaging studies, allows simple fusion of the two resulting image sets and also gives the anatomical reference to relate kidney position. The CT component also facilitates attenuation correction of the SPECT data which enables accurate quantification of regional radiotracer uptake. Sequential SPECT/CT studies can be spatially registered to planned radiotherapy dose distributions, via registration of the accompanying CT component to the CT images obtained for radiotherapy treatment simulation and planning.

**Figure 1:**
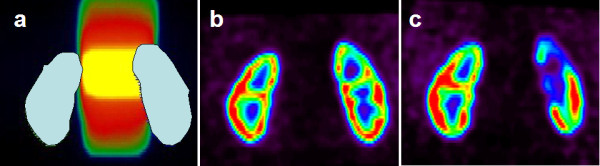
**A preliminary qualitative example.** Sample patient data from a pilot study at SCGH. The images are coronal slices, the grey shapes are the outlines of the kidneys. **a)** Planned radiotherapy dose distribution (yellow 45 Gy to black 0 Gy) and kidney volumes. **b)** Baseline (pre-treatment) SPECT intensity distribution (red - high activity, blue – low activity). **c)** 12-week post treatment SPECT showing reduced perfusion in the high-dose region.

Results from a pilot study at SGH are shown on Figure 1.

### Study hypotheses

An assessment of changes in total kidney function (using GFR measurement), regional kidney functional change (using SPECT imaging) and their correlation with radiotherapy dose delivery is within the means of currently available imaging, therapy and computational technology. The hypotheses being tested in RAPRASI are:

– Temporal variations in SPECT images of the kidneys correlate with patterns of radiation dose delivery in the kidneys.

– Models of kidney response developed from SPECT-response studies can be used to predict changes in kidney function as a result of radiation therapy.

The principal aim of RAPRASI is to obtain a better understating of kidney response when a patient is receiving radiotherapy for an upper-abdominal cancer. This will involve monitoring the spatial and temporal changes in kidney function for correlation with delivered three-dimensional radiation dose distribution. A more precise dose-volume response model for kidney will not only improve our current understanding but may permit further optimisation of radiation therapy techniques for upper-abdominal tumours.

## Methods/design

### Study design and schedule

RAPRASI is a clinical research study for participants with upper gastrointestinal malignancies (without metastatic disease) and who have controlled and stable kidney function. Radiotherapy treatment technique is according to local departmental protocols. Participants are being recruited as they present to the Department of Radiation Oncology, Sir Charles Gairdner Hospital (SCGH) in Perth, Australia, and to the Department of Radiation Oncology, Peter MacCallum Cancer Centre (PMCC) in Melbourne, Australia. The protocol has been approved by both institutional ethics committees. All patients are required to give written informed consent.

In addition to a baseline visit, timed to coincide with participants having a CT scan for the purpose of radiotherapy simulation, participants will have follow-up visits at 4, 12, 26, 52 and 78 weeks subsequent to the first radiotherapy treatment fraction. On each occasion they will undergo: a sequential ^99m^Tc-DMSA SPECT/CT (simultaneous SPECT and CT images), a GFR measurement (^51^Cr-EDTA clearance), a full blood count and assessment of blood urea and electrolytes.

The timing of SPECT/CT scans is intended to reveal i) acute and sub acute changes in total kidney function (GFR measurement) before and following radiotherapy, ii) information on kidney positioning before and following radiotherapy, and iii) the time-dependence of changes in regional kidney function and position (SPECT/CT scans) before and following treatment.

### Inclusion criteria

– Males or females older than 18 years of age.

– Histologically/cytologically proven upper GI adenocarcinoma.

– Signed written informed consent provided to participate in the study.

– Radical radiotherapy (dose > 40 Gy in 4 weeks) being prescribed to an upper abdominal site that will involve incidental radiation of one or both kidneys.

– Loco regional staging of primary disease will have been undertaken with dual phase (arterial and portal) spiral CT.

– Serum creatinine ≤150 *μmol/L.*

– Performance status is ECOG grade 0, 1 or 2 [17].

### Exclusion criteria

– Major co-morbid illnesses that, in the opinion of the investigator, would prevent the patient undergoing planned SPECT/CT scans during the first year following treatment.

– Evidence of metastatic disease.

– Significant loss of bodyweight (>15% weight loss since diagnosis).

– Inadequate bone marrow function to undergo planned therapy.

– Previous drug-induced nephrotoxicity and/or planned treatments that are likely to cause drug-induced nephrotoxicity.

– Previous abdominal radiotherapy.

– Pregnancy or lactation.

### Data collection and quality assurance

This study will be conducted according to the Human Research Ethics Committee (HREC)-approved study protocol and the ‘Note for Guidance on Good Clinical Practice (CPMP/ICH/135/95) annotated with TGA comments’ (Therapeutic Goods Administration DSEB July [18]. The study will be performed in accordance with the NHMRC ‘National Statement on Ethical Conduct in Human Research’ (© Commonwealth of Australia 2007) and the ethical principles within the ‘Declaration of Helsinki’ (Revised 1996) [19].

Sir Charles Gairdner Hospital will act as the sponsor of this study at both the Peter MacCallum Cancer Centre and Sir Charles Gairdner Hospital in Perth, Western Australia. Standard medical care (prophylactic, diagnostic and therapeutic procedures) remains the responsibility of the patient’s treating physician.

The investigator is responsible for ensuring that no participant undergoes any study related examination or investigations prior to obtaining written informed consent. The investigator will explain the aims, methods, possible benefits and potential hazards before the participant gives consent. The participant will be given sufficient time to read the documentation, understand the project and have any questions addressed prior to signing the consent form. The participants will be advised that they have the right to withdraw their consent to participate at any time without any consequences.

Participant confidentiality will be protected at all times. In any resulting publication or presentation, no patients will be identified.

Records will be retained for 15 years post study completion in a locked facility, in accordance with TGA requirements. Access to participant study records will be limited to treating clinician, chief investigator, his/her nominated co-investigators and the local study co-ordinators.

Collation of data will yield the following for each participant:

– Local kidney function/response to therapy: regional change in SPECT signal intensity:

•from baseline to 4 weeks post start of radiotherapy

•from baseline to 12 weeks post start of radiotherapy

•from baseline to 26 weeks post start of radiotherapy

•from baseline to 52 weeks post start of radiotherapy

•from baseline to 78 weeks post start of radiotherapy

– Planned 3D Regional radiation dose distributions intended to be experienced during radiation treatment.

– Kidney function measure: GFR at baseline, 4, 12, 26, 52 and 78 weeks.

– Indication of existing and acquired co-morbidities.

– Information on adjuvant treatments.

GFR measurements using the ^51^Cr-EDTA clearance and Digital Imaging and Communication in Medicine (DICOM) formatted SPECT/CT images will be obtained on Siemens Symbia T6 SPECT/CT scanners located in the Department of Nuclear Medicine at SCGH and the Centre for Molecular Imaging at the PMCC. These images involve pre-injection of approximately 185 MBq of ^99m^Tc-DMSA (Radpharm DMS 1987/1), providing 128x128 pixel attenuation-corrected images reconstructed at approximately 5 mm pixel resolution. SPECT images are automatically registered to CT taken on the same device (6 slice helical CT scanner), with image settings sufficient for organ definition and bone matching with minimal radiation dose (typically 130 kV, 80-100 mA, 5 mm slice thickness). SPECT pixel intensity values will be normalised according to calibration images obtained on the scanner during monthly quality-assurance checks. SPECT data will be reconstructed using an ordered subset expectation maximization (OSEM) algorithm incorporating CT attenuation data.

Treatment plan data (images, structures, beam definitions, dose-volume data, and 3D dose distribution) will be exported in DICOM-RT format from the Treatment Planning System. (CMS XiO at SCGH, Varian Eclipse at PeterMac). Digitally reconstructed radiographs (DRRs) will be generated indicating local bony anatomy (upper pelvis, spine, ribs) and the location of kidneys from the planning CT. Variation in kidney position relative to local bony anatomy is of the order of SPECT image resolution [20,21]. Patient localisation will initially be to external marks, with on-line positioning then corrected according to localisation images spatially matched to the DRRs. Localisation images will be obtained either with the Brainlab Exactrac system (6D patient positioning) [22] or the Varian On-Board Imaging system (3D positioning) [23]. Applied patient shifts will be recorded together with localisation images. Where possible, kidney position will be identified on the localisation images for comparison with planned kidney outlines, as per standard departmental practice.

It is anticipated that all participant data will be utilized in the final analysis provided that each participant completes at least the baseline (pre-radiotherapy) and first SPECT/CT scan at 4 weeks post start of radiotherapy. If a participant commences radiotherapy (i.e. completes the baseline SPECT/CT scan) but withdraws consent mid-treatment or is unable to complete any further SPECT/CT scans, then the data for that participant will be excluded from the final analysis.

Data relating to co-morbidities and concurrent treatments, including adjuvant chemotherapy, known to influence kidney toxicity [2], will be extracted from participant notes.

### Statistical considerations

A target of 30 complete participant datasets has been set based on an assessment of anticipated patient numbers, recruitment and attrition rates, together with a power calculation of the number of datasets required to indicate a statistically significant variation in SPECT image intensity. A clinically significant difference would be a reduction of 20% from baseline to a follow-up imaging study [5]. In order to detect a conservative estimate of a 10% reduction, where the measurement error is 20% we would require a sample size of 33 (using a paired t-test with alpha = 0.05 and power = 80%; detecting 10.5% with 30 patients which is sufficient). With an anticipated 20% non-completion/attrition rate, allowance will be made for up to 40 recruited patients. Given the intensive data analysis required per patient dataset in this study, 30 datasets represents an upper-limit in terms of manageability.

### Data analysis

**Figure 2:**
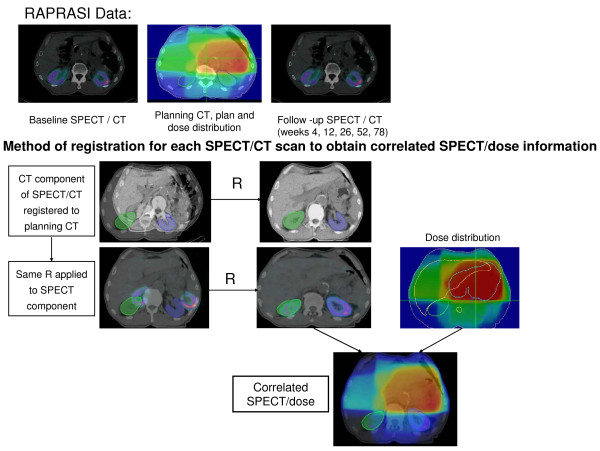
Diagram of the co-registration method.

To correlate the patterns of change in SPECT signal with time relative to delivered dose distributions, SPECT signal intensity will be used as a measure of local renal function – a change in this measure is an indication of ‘renal response’. This response needs to be correlated with regional radiation dose distribution. Planned 3D radiation dose distributions will be digitally registered with kidney functional changes in order to generate dose response models. The planning CT will be registered to the initial SPECT/CT data for definition of baseline kidney location and function. Subsequent SPECT/CT data (intensity-normalised according to administered nuclide activity) will be registered both using bone anatomy as well as SPECT intensity distribution providing registration of radiation dose distribution and SPECT signal. All imaging shall be performed with the patient in the same position on a flat-topped couch, allowing quantification of changes in kidney positioning in the abdomen relative to the planned dose distribution. Co-registrations will be made using the Velocity AI software [24]. Velocity’s deformable image registration will allow subsequent SPECT images to be deformable registered to the baseline SPECT/CT, on the basis of both CT anatomy and SPECT signal, and for all co-registered data sets to be deformable registered to the planned 3D dose distribution. This will allow subsequent SPECT images to be deformable registered to the baseline SPECT/CT, on the basis of both CT anatomy and SPECT signal, and all co-registered data sets to be deformable registered to the planned 3D dose distribution (or vice-versa), exported from the Treatment Planning System in DICOM-RT format. Applying these methods to data collected during the RAPRASI study will allow accurate spatial registration of regional changes in SPECT signal to planned radiotherapy dose. The method of registration to obtain correlated SPECT/dose is shown on Figure 2.

This subsequent analysis of co-registered dose and image data will be undertaken in several ways:

i. Methods will be developed to assess dose-response correlations as a function of spatial position across each kidney (sampled at varying resolutions). Kidney volumes will be uniformly divided into regions on the three-dimensional (3D) grid defining image and spatially-registered radiation dose. t-tests and Wilcoxon signed-rank tests will be undertaken to determine regions with significantly different mean SPECT-signal intensities between time-points for each study participant. Mixed modelling with repeated measures (and random subject effect) will be conducted across all participant sets to examine the correlation of local radiation dose with SPECT signal changes, generating a map of dose-response change across all kidney volumes. Given a significant correlation, this will be extended to incorporate any revealed patterns of co-morbidity or adjuvant therapy as categorical variables.

ii. Dose-response correlations will then be examined according to structural and functional regions. Analysis i. will be repeated, but with regions defined according to kidney structure, with medulla, renal artery and cortex regions treated independently. This will also involve cross-correlation between these regions to examine inter-play that is likely to arise due to the influence of radiation effects on vascular structures.

iii. Spatio-temporal mathematical models of functional kidney dose-response will be implemented to relate regional kidney irradiation to the primary measure of total kidney function, GFR. These models, including percolation theory [25] and the critical functioning volume model of Rutkowska et al [26], will utilise the 3D radiotherapy dose distribution (both across each kidney and across individual functional regions) to fit model parameters using maximum likelihood estimation (MLE) methods. The statistical assessment of fitting will be made by assessment of parameter confidence intervals, and by undertaking an F-test of the residual sums-of-squares from MLE against that from MLE for a simple linear (single-parameter) dose-response model.

iv. An export of co registered dose and SPECT information will be made for regions defined by whole kidneys, separately-identified medulla and cortex, baseline-image functional regions, and at varying spatial resolutions. Renal response patterns obtained from variations in SPECT and GFR shall be fitted to normal-tissue complication probability (NTCP) and equivalent uniform-dose (EUD) models, as previously undertaken by CI’s using estimated parameters [27,28]. Response indicators can be related to dose volume histogram (DVH) and dose-function histogram (DFH) data by maximum likelihood fitting to the parametric NTCP/EUD models. For this purpose, the freely available DREES code [29], developed in Matlab, will be utilised. Separate analyses will be undertaken using acute changes in SPECT signal (< 12 week images) as well as the long-term changes indicated by images taken at 52 or 78 weeks post-radiotherapy.

Examination of the predictive nature of response models will be made by establishing parameters based on the first 20 patient data sets, and utilising the models to predict SPECT signal changes on the subsequent 10 patients. In order to minimise parameter uncertainties however, final parameter values will be based on data for all patients.

### Ethics

The RAPRASI study has ethics approval from the HREC at Sir Charles Gairdner Group, The University of Western Australia in Perth, and the Peter MacCallum Cancer Centre in Melbourne, Australia. The trial is registered with the Australian New Zealand Clinical Trials Registry: ACTRN 12609000322235.

## Discussion

RAPRASI is a unique study combining functional radiological imaging with radiotherapy dose delivery for the assessment of regional radiation-induced kidney toxicity. The study focuses on a patient cohort which is poorly represented in clinical studies and for whom outcomes are known to be poor. Modern radiation therapy utilises intensive calculation processes and digitally driven beam delivery technologies to sculpt dose distributions in ways that were not previously possible. To take proper advantage of this technology it is necessary to understand how normal tissues are expected to respond to highly variable radiation dose distributions and how radiation dose may be sculpted to existing functional regions without detriment to the patient or the desired tumour dose. The information gained from the RAPRASI study, including models that allow prediction of temporal changes in kidney toxicity, will add to our current understanding of partial kidney radiation dose response to improve outcomes (including maximizing the preservation of function) of kidney dose response and tolerance to radiation.

## Competing interests

The authors declare that they have no competing interests.

## Authors’ contributions

MAE, NS, PR, JB, TL, DW and SB planned and developed the study protocol. At SCGH, JLG is the study coordinator and with MAE, NS, PP and GW are responsible for patient recruitment; PR, JB and BO are in charge of conducting and reviewing the SPECT/CT scans. At PMCC JF is the research coordinator and with TL, JC, LW, are responsible for patient recruitment. MH conducts and reviews the SPECT/CT scans. JLG is responsible for data quality assurance and cleaning, image processing and final statistics analysis. All authors read and approved the final manuscript.

## Pre-publication history

The pre-publication history for this paper can be accessed here:

http://www.biomedcentral.com/1471-2407/13/381/prepub

## References

[B1] Emami B Lyman J Brown A Cola L Goitein M Munzenrider JE Shank B Solin LJ Wesson M Tolerance of normal tissue to therapeutic irradiation Int J Radiat Oncol Biol Phys 1991 21 1 109 122 10.1016/0360-3016(91)90171-Y 2032882

[B2] Dawson LA Kavanagh BD Paulino AC Das SK Miften M Li XA Pan C Ten Haken RK Schultheiss TE Radiation-associated kidney injury Int J Radiat Oncol Biol Phys 2010 76 3, Supplement 1 S108 S115 10.1016/j.ijrobp.2009.02.089 20171504

[B3] Thompson PL Mackay IR Robson GSM Wall AJ Late radiation nephritis after gastric x-irradiation for peptic ulcer QJM 1971 40 1 145 157 5090542

[B4] Willett CG Tepper JE Orlow EL Shipley WU Renal complications secondary to radiation treatment of upper abdominal malignancies Int J Radiat Oncol Biol Phys 1986 12 9 1601 1604 10.1016/0360-3016(86)90284-1 3759586

[B5] Kost S Dorr W Keinert K Glaser FH Endert G Herrmann T Effect of dose and dose-distribution in damage to the kidney following abdominal radiotherapy Int J Radiat Biol 2002 78 8 695 702 10.1080/09553000210134791 12194753

[B6] Jansen EPM Saunders MP Boot H Oppedijk V Dubbelman R Porritt B Cats A Stroom J Valdés Olmos R Bartelink H Prospective study on late renal toxicity following postoperative chemoradiotherapy in gastric cancer Int J Radiat Oncol Biol Phys 2007 67 3 781 785 10.1016/j.ijrobp.2006.09.012 17157445

[B7] Robbins MEC Campling D Rezvani M Golding SJ Hopewell JW Radiation nephropathy in mature pigs following the irradiation of both kidneys Int J Radiat Biol 1989 56 1 83 98 10.1080/09553008914551211 2569012

[B8] Robbins MEC Bywaters T Rezvani M Golding SJ Hopewell JW The effect of unilateral Nephrectomy on the subsequent radiation response of the pig kidney Int J Radiat Biol 1991 59 6 1441 1452 10.1080/09553009114551291 1677388

[B9] Levey AS Eckardt KU Tsukamoto Y Levin A Coresh J Rossert J de Zeeuw D Hostetter TH Lameire N Eknoyan G Definition and classification of chronic kidney disease: a position statement from kidney disease: improving global outcomes (KDIGO) Kidney Int 2005 67 6 2089 2100 10.1111/j.1523-1755.2005.00365.x 15882252

[B10] Cohen EP Robbins MEC Radiation nephropathy Semin Nephrol 2003 23 5 486 499 10.1016/S0270-9295(03)00093-7 13680538

[B11] Dawson LA Biersack M Lockwood G Eisbruch A Lawrence TS Ten Haken RK Use of principal component analysis to evaluate the partial organ tolerance of normal tissues to radiation Int J Radiat Oncol Biol Phys 2005 62 3 829 837 10.1016/j.ijrobp.2004.11.013 15936567

[B12] Robbins ME Brunso-Bechtold JK Peiffer AM Tsien CI Bailey JE Marks LB Imaging radiation-induced normal tissue injury Radiat Res 2012 177 4 449 466 10.1667/RR2530.1 22348250 PMC3733443

[B13] Daly MJ Jones W Rudd TG Tremann J Differential renal function using technetium-99m dimercaptosuccinic acid (DMSA): in vitro correlation J Nucl Med 1979 20 63 66 219166

[B14] Peters AM Scintigraphic imaging of renal function Exp Nephrol 1998 6 5 391 397 10.1159/000020547 9730654

[B15] Robins P Boucek J Bydder S Osborne C Podias P Ebert MA Spry N Co-registration of renal SPECT scans with treatment planning data to refine radiobiological understanding: a true marriage of radiology and radiotherapy 2004 Perth WA: 2004 Scientific Meeting of the Royal Australasian and New Zealand College of Radiologists 2004

[B16] Even-Sapir E Keidar Z Bar-Shalom R Hybrid imaging (SPECT/CT and PET/CT)-improving the diagnostic accuracy of functional/metabolic and anatomic imaging Semin Nucl Med 2009 39 4 264 275 10.1053/j.semnuclmed.2009.03.004 19497403

[B17] Oken MM Toxicity and response criteria of the eastern cooperative oncology group Am J Clin Oncol 1982 5 6 649 655 10.1097/00000421-198212000-00014 7165009

[B18] Note for guidance on good clinical practice (CPMP/ICH/135/95) annotated with TGA comments http://www.tga.gov.au/pdf/euguide/ich13595.pdf

[B19] National statement on ethical conduct in human research http://www.nhmrc.gov.au/_files_nhmrc/publications/attachments/e72_national_statement_130207.pdf

[B20] Li XA Qi XS Pitterle M Kalakota K Mueller K Erickson BA Wang DA Schultz CJ Firat SY Wilson JF Interfractional variations in patient setup and anatomic change assessed by daily computed tomography Int J Radiat Oncol Biol Phys 2007 68 2 581 591 10.1016/j.ijrobp.2006.12.024 17331669

[B21] Van Der Geld YG Senan S De Koste J Verbakel W Slotman BJ Lagerwaard FJ A four-dimensional CT-based evaluation of techniques for gastric irradiation Int J Radiat Oncol Biol Phys 2007 69 3 903 909 10.1016/j.ijrobp.2007.06.062 17889271

[B22] Jin JY Yin FF Tenn SE Medin PM Solberg TD Use of the BrainLAB exactrac X-ray 6D system in image-guided radiotherapy Med Dosim 2008 33 2 124 134 10.1016/j.meddos.2008.02.005 18456164

[B23] Mechalakos JG Hunt MA Lee NY Hong LX Ling CC Amols HI Using an onboard kilovoltage imager to measure setup deviation in intensity-modulated radiation therapy for head-and-neck patients J Appl Clin Med Phys 2007 8 4 28 44 10.1120/jacmp.v8i4.2439 PMC5722619 18449150

[B24] VelocityAI: velocity medical solutions: radiation oncology PACS : deformable image registration http://www.velocitymedical.com/velocityai

[B25] Thames HD Zhang M Tucker SL Liu HH Dong L Mohan R Cluster models of dose-volume effects Int J Radiat Oncol Biol Phys 2004 59 5 1491 1504 10.1016/j.ijrobp.2004.04.001 15275737

[B26] Rutkowska E Baker C Nahum A Mechanistic simulation of normal-tissue damage in radiotherapy-implications for dose-volume analyses Phys Med Biol 2010 55 8 2121 2136 10.1088/0031-9155/55/8/001 20305336

[B27] Spry N Harvey J MacLeod C Borg M Ngan SY Millar JL Graham P Zissiadis Y Kneebone A Carroll S 3D radiotherapy can be safely combined with sandwich systemic Gemcitabine chemotherapy in the management of pancreatic cancer: factors influencing outcome Int J Radiat Oncol Biol Phys 2008 70 5 1438 1446 10.1016/j.ijrobp.2007.08.070 18164859

[B28] Osborne C Bydder SA Ebert MA Spry NA Comparison of non-coplanar and coplanar irradiation techniques to treat cancer of the pancreas Australas Radiol 2006 50 5 463 467 10.1111/j.1440-1673.2006.01627.x 16981944

[B29] El Naqa I Suneja G Lindsay PE Hope AJ Alaly JR Vicic M Bradley JD Apte A Deasy JO Dose response explorer: an integrated open-source tool for exploring and modelling radiotherapy dose-volume outcome relationships Phys Med Biol 2006 51 22 5719 5735 10.1088/0031-9155/51/22/001 17068361

